# Work as Meaningful and Menacing Phenomenon for South African Middle Managers During the COVID-19 Pandemic: The Role of Self-Transcendence in Cultivating Meaning and Wellbeing

**DOI:** 10.3389/fpsyg.2021.650060

**Published:** 2021-06-16

**Authors:** Aden-Paul Flotman

**Affiliations:** Industrial and Oranisational Psychology, University of South Africa, Pretoria, South Africa

**Keywords:** COVID-19 pandemic, employee wellbeing, existential positive psychology, meaningful mindset, reflexivity, self-transcendence

## Abstract

Self-transcendence has become and remains an important research theme. Little is known about the role of self-transcendence in cultivating meaningful work and its impact on the wellbeing of middle managers in the face of adversity, such as the COVID-19 pandemic. The aim of this qualitative hermeneutic phenomenological study was to explore the impact of the COVID-19 pandemic on the meaning middle managers attach to their work by investigating the role of self-transcendence in cultivating meaning and wellbeing in a cohort of seven South African middle managers employed in cross-boundary service industry settings. Data were collected through unstructured narratives. Findings confirm that self-transcendence serves as a coping mechanism during adversity and that it facilitates the re-negotiation of meaning, resulting in three potential shifts: the shift from a blame orientation to a work orientation, the shift from reflection to reflexivity and the shift from self-consciousness to other-consciousness. The findings also highlight how self-transcendence enables the exploration of the adaptive benefits of anxiety. The findings contribute new insights into the construct of self-transcendence and extend research on existential positive psychology. It is suggested that organizations invest in reflexive practices as a tool to promote deep learning and connectivity by exploring dialectical processes through reflexive work.

## Introduction

*“Lockdown”* is officially the South African word for 2020. I write this article as the second wave of the COVID-19 pandemic continues to make itself felt across Europe and the United States. As the pandemic unfolds, it persistently presents unprecedented social, political and economic challenges. Individuals and groups continue to face increasing concern about coping with their prevalent anxieties and the long-term individual and collective impact of the pandemic (Koenig, 2020; Peteet, 2020). What is increasingly noticed is how the pandemic is affecting individuals to the core and the subsequent release of deeply human emotions (Modell and Kardia, 2020).

In informal conversations the infamous words in the Charles Dickens novel, *A Tale of Two Cities*, are often cited: “It was the best of times, it was the worst of times … it was the season of Light, it was the season of Darkness. It was the Spring of hope, it was the Winter of despair …” (Dickens, 1997, p. 13). This passage, though written in a different context, is also applicable today. The pandemic has revealed the best of humanity, but unfortunately, also continues to expose the worst of humanity. We have witnessed the excessive stockpiling of food and personal amenities, criticism about how in hindsight, the pandemic should have been managed and the increasing revolt against stringent lockdown regulations. In this context, the dark side of our human existence is revealed (Wong, 2009). As we negotiate this crisis, one of the things it has emphasized is that we need to accept accountability in our own space. Evidence of this brave human response manifested in medical staff risking their lives to attend to the sick and frail. Even social media are littered with reports on how neighbors as well as strangers reach out to the less fortunate through random acts of kindness.

Despite these positive developments, the impact of the pandemic is poised to reverberate into the foreseeable future across our social, economic and political landscape. COVID-19 continues to challenge and change everything. This includes the inadequacy of certain psychologies. The pandemic, like a cancer, has evoked in us deeply rooted existential anxieties (Pandya, 2019; Peteet, 2020). In evoking these anxieties, the pandemic presents a threat to what is considered normal, our personal identities and our sense of place in the world (Kaufman, 2020). Furthermore, individuals and societies are being confronted with existential questions, including challenges to their fundamental assumptions about the world. Many of these questions trigger psychological provocations, in particular about life as a site of struggle and the meaning of suffering, as espoused by existential positive psychology. As we reflect on our brokenness and lament our suffering, it is argued that existential positive psychology is a useful theoretical lens with its unequivocal embrace of suffering as an inevitable human phenomenon (Vozza, 2020) and the subsequent search for flourishing by being mindful of the ever-present constraints of reality (Lomas and Ivtzan, 2016; Apter, 2020; Wong, 2020).

In the context of suffering and adversity, meaningfulness has an impact on individual, psychological and work-related outcomes (Haidt, 2013; Mayer et al., 2015; Wong and Bowers, 2018). This is also applicable to the participants in this study, namely South African middle managers in cross-boundary service industry settings. The primary role of middle managers is to allocate human, material and information resources in pursuit of an organization’s goals (Keeman et al., 2017, p. 510). This role of leading the organization to success has become an even more difficult task during the COVID-19 pandemic, which turned out to be one massive change management process. The service industry was one of the hardest hit industries in the economy. The hard lockdown resulted in businesses closing, some permanently. Most employees had to work from home, which was for many a new reality to get used to. Managers had to set employees up to be able to work from home and even more importantly, manage and lead them remotely. The absence of the intimacy of the office was another challenge for many employees. This complex and dynamic situation inevitably affected employees’ and managers’ wellbeing (Khoury, 2020; Modell and Kardia, 2020) and the meaning they attached to their work during this time (Martela et al., 2018; Bartels et al., 2019). Meaningfulness at work has been reported as a critical psychological condition (Van Zyl et al., 2010). Recent studies have concentrated on the role of positive affect (King et al., 2006), challenges of role identities (Sterley, 2014), meaningfulness of work for women in higher education (Mayer et al., 2015), and meaningfulness as satisfaction of autonomy, competence, relatedness and beneficence (Martela et al., 2018). However, little is known about the role of self-transcendence (ST) in cultivating meaningful work and its impact on the wellbeing of middle managers during hardship and misfortune. Thus, the objective of the study was to gain in-depth understanding of the meaning middle managers attach to their work in the face of adversity, such as the COVID-19 pandemic. This is done by investigating the role of ST in cultivating meaning and wellbeing. It is evident from previous studies that there is an association between the capacity to be other-centered (as embodied in ST, meaningful work and ultimately wellbeing (Lomas and Ivtzan, 2016; Person et al., 2016; Bartels et al., 2019). The study contributes to new insights into the construct of ST in relation to existential positive psychology. Furthermore, it confirms that ST could be used to harness the adaptive benefits of anxiety and affirms that ST plays a significant role in building psycho-social resilience, relatedness and character.

In the next section, the theoretical framework of the article is provided, followed by the purpose and aim of the study and the research methodology.

## Theoretical Perspectives

### Meaningful Life, Work, and Wellbeing

The literature on meaningfulness affirms that the ability to work continues to play a critical life role (Lambert et al., 2013; Rothmann and Hamukang’andu, 2013; Van Tongeren et al., 2015), which results in one becoming a functional adult (Gilligan, 1997; Bakar et al., 2018). Work plays a socially integrative function to determine one’s status, social prestige and connectedness to significant others (Ransome, 1996). It has also been reported that identity and a coherent sense of self is developed through meaningful work and positive relationships (Van Wyk, 2012; Person et al., 2016). Often one’s search for meaning in personal existence happens through work (Frankl, 1959; Bartels et al., 2019). Thus, work has been defined as an action that results in a service of value or value in the form of goods for others (Grossman and Chester, 2013). In doing work, one’s need for meaning and connectedness is satisfied (Keeman et al., 2017). These findings have value in the socio-economic and political context of this study, because during the COVID-19 pandemic, employees were severely restricted in terms of the nature of their work and their ability to interact with their colleagues. However, these restrictions also presented new opportunities in the form of reaching out to others.

The construct of meaningfulness is an expression of one’s subjective assessment of life, the attributed significance of life events and the subsequent identity that is created through these interactions (Matuska and Christiansen, 2008; Bakar et al., 2018). Because individual meaningfulness, beliefs and values are expressed through work, work becomes meaningful. Meaningfulness at work is therefore a critical psychological condition in pursuit of personal development, growth and self-actualization (Durbin and Tomlinson, 2014). Work is meaningful when it is inherently valued, particularly when it is congruent with one’s personal value system and competence (Martela et al., 2018). Four predictors of meaning stand out in the literature, namely autonomy, competence, relatedness and beneficence (Steger, 2012; Kun and Gadanecz, 2019). The pandemic seemed to have significantly eroded autonomy, competence (to some extent) and relatedness. However, beneficence seemed to have emerged as a significant leveraging device.

It is not surprising that meaning in life has been associated with salient components of human wellness (Wong, 1989). Research indicates that employees who experience high levels of wellbeing are those who put a lot of effort into their work, experience fewer work injuries, are absent from work less often and are committed to the organization (Keeman et al., 2017; Bartels et al., 2019). Wellbeing is defined as a “dynamic, extensive and contextual self-appraisal made by an individual when evaluating their life in terms of their potential, aspirations, values and apprehensions …” (Dodge et al., 2012). The result of wellbeing is social, physical, mental and environmental health. This kind of health is experienced when there is congruence between one’s thoughts, values and actions, leading to self-understanding, autonomy, a sense of achievement and satisfaction with life (Deci and Ryan, 2008).

### Self-Transcendence and Wellbeing

Research seems to indicate that ST is a basic psychological need (Ryan and Deci, 2000) and a fundamental human motivation (Levenson et al., 2005). When we give our best to be in service of others, this new way of being is called ST (Wong, 2016). It has also been suggested that ST is connected to beneficence—a sense of influencing the lives of others in a positive manner, which has also been associated with wellbeing (Martela and Ryan, 2016). Thus, lived experiences of beneficence in the form of caring for others have become a major component of meaning and wellbeing (Van Tongeren et al., 2015). When ST is exercised, one contributes to a cause bigger than oneself, which often results in a meaningful life (Frankl, 1963; Wong, 1989; Van Tongeren and Van Tongeren, 2020). Meaningfulness emanates from ST in the form of reaching out and connecting with other people and the external world (Weinstein et al., 2012). The precondition for this is ST, which is key for not just survival, but human flourishing as well (Runquist and Reed, 2007).

According to Wong (2016), three levels of meaning-seeking lead to ST. These levels are “meaning of the moment through mindful awareness; meaning in life through the pursuit of a calling; and ultimate meaning through faith in a transcendental realm” (p. 311). In a sense, ST redirects our focus to become other-conscious. Being human points to something beyond the self (Hicks and Routledge, 2013). This is a very clear reminder of the African concept of “*ubuntu*,” which literally means that a person is a person through other people (Mwipikeni, 2018). Thus, human beings are relational beings. We become human when we realize that we are incomplete when in isolation and that we become not only human, but *fully* human when we are connected to others. Practicing ST helps people cope with inevitable disappointment because of egotism and sometimes self-destruction in pursuit of unbridled personal success and ambition. It is through ST that we experience a sense of belonging, fulfillment and wellbeing. Pope Francis echoes these sentiments when he writes:

*Kindness frees us from the cruelty that at times infects human relationships, from the anxiety that prevents us from thinking of others, from the frantic flurry of activity that forgets that others also have a right to be happy* (Pope Francis, 26 October 2020).

### Reflexivity, Agency, and a Meaningful Mindset

Reflexivity seems to be key in the context of creating a meaningful mindset and to enhance one’s sense of purpose and wellbeing (Marginson, 2014). The consistent significance of reflexivity is evident from the growing body of research on the topic. Reflexivity has been studied and conceptualized in different ways (Pillow, 2003; Helyer, 2015; Matthews, 2017). Reflective practice comprises two related processes, namely reflection and reflexivity (Fergusson et al., 2019). Self-awareness seems to be a key component of reflection (Rennie, 1992). Self-reflexivity, on the other hand, entails an ongoing meta-level reflective process (questions, thoughts and feelings) about the self and how this reflective process is being consciously experienced in the moment (Nagata, 2004). Archer (2003) conceptualizes reflexivity as an internal conversation. In this conversation, individuals reflect on the following: “what’s going on?” and “what am I going to do?” This is an ongoing “reflexive project” as individuals reflect upon and monitor themselves (Giddens, 1984). In sum, reflexivity is the process of recognizing what is happening explicitly and implicitly in the moment (Engward and Davis, 2015). This deeper form of thinking and learning enables sense-making and meaning-making particularly during a pandemic.

It has been argued that reflective practice should result in agency (Fergusson et al., 2019, p. 12). Agency is conceptualized as the capacity of individuals to control the nature and quality of their lives (Bandura, 2001). Furthermore, agency is always positioned in relation to a given context. As one’s context changes, so agency should change. This is important because individuals must contend with different realities, challenges and possibilities (Matthews, 2017). In the final analysis, individuals must actively reflect on their lives—meaningful mindset—as part of an enduring “reflexive project.” This discussion is important in the context of this study because reflexivity should result in the realization of one’s own power and authority, particularly during difficult times. In realizing one’s personal agency, one should realize that a positive difference can be made in other people’s lives. This other-orientation should enhance personal wellbeing, which is the active pursuit of choices and lifestyles that relate to one’s purpose and calling in relation to others (Deci and Ryan, 2008).

## Materials and Methods

This article reports on the phenomenological experiences of participants during the hard lockdown in South Africa during the COVID-19 pandemic. The next sections present a discussion on the research methodology and an explication of the research setting and conclude with the participants, data protocols and the data analytic strategy that directed the study.

### Research Methodology

The nature of this study was exploratory and descriptive. A qualitative inquiry in the psycho-social tradition was deemed most appropriate to explore and describe the lived experience of participants (Laverty, 2003). Consequently, the researcher selected hermeneutic phenomenology to direct the design of the study. Hermeneutics means working on the text for its truth to emerge (Gadamer, 2004). Phenomenology is the study of how things show or give themselves (Marion, 2002). Therefore, in its rudimentary form, hermeneutic phenomenology is concerned with the life world or human experience as it is lived (Van Manen, 2017) and implies working with part and whole in a cyclical, open and interrogative manner, to understand the *participants* who produced the text, the *person* doing the hermeneutic phenomenological work, and the *phenomenon* that is brought to awareness and made manifest as a result of the work (Suddick et al., 2020a, p. 12). This design is applicable to this study, because in hermeneutic phenomenological studies, meaning and understanding is co-constructed by both the participants and the researcher (Lauterbach, 2018). The transparency and authenticity of the researcher’s pre-understanding is explored, rather than no-bias and objectivity (Gentles et al., 2015). This design was also relevant because it presupposes that knowledge is relative to the participants (their subjective experiences of the pandemic), the research context and the researcher’s preconceptions (Bu and Paré, 2018). In its approach and design, this study therefore allowed for the explication of participants’ lived experience and in-depth understanding of the meaning middle managers attach to their work in the face of adversity.

### Research Setting

The research setting of this study is cross-boundary service industries. Participants came from service settings ranging from retail to educational institutions. All the participants reflected on and recorded their experiences in their own time and in their own space according to their specific unique circumstances. Because of the volume of the data, the software statistical package Atlas.ti was used to store and later to process the data.

### Data Protocols

Data were collected through seven unstructured narratives, reporting on the lived experiences of middle managers during the pandemic. Seven participants volunteered to record their experiences in the form of journal reflections (August to September) and submit their narratives by the first week of October 2020. The main question was open-ended to guard against being prescriptive and to elicit rich data to explore the life world of the participants. The question was: *“I would like to know more about your experiences as a middle manager. Please tell me about your wellbeing experiences during this time of the pandemic. What is it like to be a middle manager during this time?”* Thus, the main question was formulated in such a way that it was not directive, to guide critical self-reflection in line with the objective of the study. The final reflective narratives were written in conversational style as participants shared their life-world experiences in a story-telling format (Goble, 2016; Dahlberg and Dahlberg, 2020) until the phenomenon being studied was brought to awareness (Suddick et al., 2020b).

### Research Sampling and Participants

A convenient, purposive sampling strategy was employed to select relevant participants for the study. Participants were included in the sample based on their role as line managers, their relevant working experience as directed by the purpose of the study and their willingness to share their working and wellbeing experiences during the COVID-19 pandemic. Eventually, seven participants with the willingness to share their information-rich experiences volunteered to participate in the study. In terms of phenomenological research, these seven participants constituted an adequate sample size (Smith and Shinebourne, 2012; Gentles et al., 2015; Churchill, 2018). The socio-demographic profiles of the participants are presented in Table 1 below.

**TABLE 1:** Participant profiles.

**Participant**	**Gender**	**Population**	**Employment**	**Role**	**Work**
					**Sector**
PR1	Male	Black	Part-time	Middle manager	Retail
PR2	Female	Black	Full-time	Middle manager	Education
PR3	Female	Black	Full-time	Middle manager	Consulting
PR4	Female	White	Full-time	Middle manager	Retail
PR5	Male	Black	Full-time	Middle manager	Education
PR7	Female	Black	Full-time	Middle manager	ITC
PR9	Female	Black	Part-time	Middle manager	Consulting

### Ethical Considerations

Ethical approval had to be obtained to conduct the research study. Approval was obtained from the University of South Africa’s (UNISA) Ethics Review Committee with reference number (REF#:2019_CEMS/IOP_040). Participants were fully informed about the nature of the study and the possibility of the data being used for research purposes. They consented in writing that the data could be used by the researcher. As a registered industrial and organizational psychologist, the researcher is expected to abide by the ethical code of conduct for psychologists as promulgated by the Health Professions Council of South Africa. In his role as an academic, the researcher had to adhere to UNISA’s ethics policy. To ensure anonymity, pseudonyms are used to protect the privacy of the participants. Furthermore, ethical principles of confidentiality, informed consent and no harm to participants were upheld by reporting on the data in a narrative, collective and thematic manner. Thus, the study was conducted in accordance with the basic principles espoused in the Declaration of Helsinki.

### Data Analysis

Data were analyzed through hermeneutic phenomenological analysis. This was done according to the analytic stages of naïve reading, structural thematic analysis and comprehensive understanding, as espoused by Lindseth and Norberg (2004). Before the data could be analyzed, a hermeneutic phenomenological attitude had to be adopted (Gadamer, 2004), because the adoption of a phenomenological attitude is necessary to lay claim to any form of phenomenological research (Giorgi, 2009). In this study it involved making visible any fore-meaning; being open and flexible by adopting a questioning attentiveness to what enters one’s consciousness; directing rigorous attention to how any meaningful lived experience “echos” in and through the body; and engaging in a play-process of hermeneutic dialogue, interpretation and interaction between the researcher, participants and the phenomenon (Finlay, 2014; Suddick et al., 2020b). I considered each participant’s unique meaningful experience and then in relation to the meaning constructed from their collective experience (collective whole), in an iterative manner (Crowther et al., 2016). This process also implied making use of free creative associations to determine what the data said about the phenomenon being explored (Kafle, 2013). Having compared the themes and findings across the three stages, no further new themes emerged to enrich the description of the meaning middle managers attach to their work by investigating the role of ST in cultivating meaning and wellbeing.

## Findings

The naïve reading of the texts seems to indicate that ST has played a significant role in the sense-making and meaning-making activities of participants. The second stage of the data analysis process in the form of structural analysis of the themes enabled the researcher to synthesize the enabling role of ST. Three major themes emanated from the structural thematic analysis of the data to describe the role of ST in cultivating meaning and wellbeing. The first theme describes how ST could serve as coping mechanism during adversity. The second theme describes how ST facilitates the re-negotiation of meaning, leading to potential shifts from a blame orientation to a work orientation, from reflection to reflexivity and from self-consciousness to other-consciousness. The third theme describes how ST enables the exploration of the adaptive benefits of anxiety. These three themes and associated sub-themes are conceptualized and critically discussed below. Italics are used to reflect the verbatim data from the different narratives of participants. This technique is used to illustrate the origin of the themes and to provide insight into the sense-making process during data analysis.

### Self-Transcendence Serves as Coping Mechanism During Adversity

The narratives and experiences of the middle managers reveal how being self-transcendent could serve as a coping mechanism during times of adversity, as characterized by the COVID-19 pandemic. ST as coping mechanism is demonstrated by PR9 when she says: *“*… *I was forced to exhibit courageous leadership to care for and provide direction for my staff. This provided me with a sense of purpose. Paradoxically, having identified a purpose during adversity, helped me to cope as well*…*”* Similarly, PR7 makes her coping through being other-centered in the face of adversity evident as she describes how she felt out of her depth during the pandemic as a senior leader in the organization: *“*… *The COVID-19 pandemic has challenged us greatly and it has been a short few weeks with a lot of adjustments. The social wellbeing part of my life has been disturbed. In being stretched, I was able to cope because I found strength in the collective to not only survive but flourish as well.”* These managers had to focus on others, in the form of their employees, in order to help themselves cope by finding value in their new tasks and ultimately meaning in the new, different role they had to take up. Another participant looked at the pandemic through her spiritual lens. This interpretation also resulted in her being able to cope better under challenging conditions. She (PR4) narrates: *“*… *In my experience, individuals develop a sense of spirituality throughout their lives. People who are more spiritual are able to see and experience a deeper sense of meaning. This generates resilience and a sense of hope. When I elevate myself beyond the physical, I elevate myself from the desires of the ego, my drive for self-preservation, and self-importance and having things “my way.”* Being self-transcendent and believing in the presence of a higher deity helped this manager to cope with the uncertainty, isolation and anxiety. Thus, meaning and insight can be extracted from pain, suffering and hardship. These sentiments were echoed by another middle manager (PR5) when he stated: *“*… *In order to deal with the mayhem, I had to turn to a higher deity in the form of my God and in this conversation, I had to question the purpose and meaning of life*…*”* This spiritual tone was continued by PR1: *“*… *This pandemic has confirmed that we are spiritual beings, rather than religious beings and that as human beings we are able to adapt to our external environment. Unfortunately, we only realize this when we [are] faced with hardship and adversity.”*

In addition to realizing the value of ST, middle managers also seem to affirm that personal agency is to be facilitated through ST by reaching out to others, in caring for employees and in recognizing new opportunities. In acknowledging their agency, they acknowledge that value is to be found in pain and suffering, but also that one is not relegated to be a paralyzed victim in the face of adversity. Here, we are reminded of the courage of health care workers during the pandemic, who continue to serve patients, irrespective of the danger to themselves and their loved ones.

This theme reflects how ST could serve as a coping mechanism during hardship. It also demonstrates that in being self-transcendent one can control the quality and direction of one’s life (agency). In doing this, personal resilience could be improved in the interest of enhanced wellbeing and a meaningful life.

### Self-Transcendence Facilitates the Re-negotiation of Meaning, Leading to Potential Shifts in Mental Orientation

In the narratives of these middle managers, they voiced how ST is a psychological need and human motivation, particularly in adversity, and how it has the capacity to facilitate the negotiation and re-negotiation of meaning. Meaningfulness stems from ST by reaching out to others and connecting with the other as well as the external world. PR1 explains how he felt on a daily basis a few weeks into the lockdown: *“*… *The most severe impact has been on a personal level. The virus has caused me lots of stress, anxiety and less restful sleep. I am experiencing a sick soul. It feels as if nothing matters. My spirituality has been neglected during the outbreak, because of the decline of organized religion. I am experiencing a weariness of my heart and there is nothing to fill this void.”* As an antidote to the feeling that *nothing matters*, ST, as a new way of being, places one in a position to re-negotiate meaning in an ever-changing, dynamic environment. In terms of creating meaning in an adverse context, ST seems to create potential mind-shifts.

The narratives of middle managers report a significant shift from a blame orientation to a work orientation. This purported shift from a blame to a work orientation is demonstrated by PR2 when she explains: *“*… *When a person is tested and confirmed to be infected, they have to be placed in quarantine so that they do not infect others. This can lead to emotions such as loneliness, sadness and agitation. It is easy to start blaming others, my manager, the government, the Chinese*… *However, one soon realizes that blaming helps no one. Similarly, as a leader in this pandemic context. I have to ask myself, ‘X [participant’s name], what are you going to do in this situation?”’* Being preoccupied with one’s personal circumstances is not a credible solution to any situation. The narrative indicates that one must remove oneself from the situation (being self-transcendent by creating distance), getting into work mode or action mode and focus on arriving at a sustainable solution to the imminent challenge. Other middle managers also reported on this shift to a work orientation. For example, PR4 said: *“*… *I soon realized that this current lockdown is part of my current existential reality. To counter the negative impact, I had to implement strict house rules, schedule daily routines, exercise time, study time, private time. This situation also presented opportunities in the form of more quality time with the family*…*”* Similarly, PR6 responded: *“*… *in these fear-producing events, I have to abandon my victim mentality*…*”* PR5 described how the pandemic had affected her management style: *“*… *with technology becoming the new normal, I had to stop complaining and quickly had to adapt my preference for face-to-face communication to a more visible digital presence*…*”* Another respondent (PR9) indicated that she had been *“*… *(forced to) attend lots of online courses because there was an immediate shift by the business to a more aggressive online presence*…*”* In a sense, ST creates an expansive vision. One observes what has been invisible before. This contrasts with playing the blaming game. The victim takes center stage, because blaming involves making a moral judgment that is both cognitive and social in character. Furthermore, it regulates social behavior. In doing this, agency is taken away, because the victim is on the receiving end of the pandemic.

The narratives of middle managers also report a significant shift from simple reflection to reflexivity, which is defined as a deeper form of learning as well as an enduring meta-level reflective process about the self and how it is being experienced in the moment (Nagata, 2004). This shift is reflected in the following narrative by PR1: *“*… *Now that COVID-19 has taken control of our lives, it is becoming a serious threat to our lives, our livelihood and our wellbeing. However, during the lockdown pause, as a leader I have come to the realization that it cannot be business as usual, even my management style cannot be rigid. It has to adapt, because my direct reports are looking at me for reassurance during these perilous times. I was able to gain this insight by looking at the situation from the perspective of my employees*…*”* PR2 supports this sentiment by saying that *“I looked at the pain of others and said to myself that this is a time to be of service to family, friends and others and I have to visibly demonstrate this through [a] generous spirit*…*”* Through the reflexive opportunity presented by ST PR3 even came to rediscover her African identity: *“I am in a sense appreciative of the silence of the lockdown. As an African and an African parent, I have been reflecting on the concept of wellbeing, which I often experience as ambiguous in my western corporate setting. I want to extend myself by challenging my own bias*… *I feel blessed though for having gained a different, other-perspective*…*”*

Similarly, for PR4 a sense of connectedness to the other gave her a sense of purpose and enhanced her overall wellbeing. She describes her experience as follows: *“*… *During the lockdown there were so many articles and reports on the suffering of others, which filled me with compassion. I then started to focus less on myself and more on what friends, family, my colleagues, were experiencing during these turbulent times. By reaching out to them I felt whole, complete*… *I was adding value to their lives*…*”* Another respondent came to the realization that when we go through a collective crisis it is important to realize that others could be worse off and that we are affected differently. PR3 reflects: *“*… *Initially, the impact of the pandemic was just on me, me. It then slowly dawned on me that everybody is scared and uncertain about the future, all of us have to be ready to change our lives, all families, children and employees have to be reassured that all would be well. At least I still have a job, a vehicle, money in my account, relevant skills*…*”* PR5 found solace through the insight that he *“had to be careful what he reads and watches*…*”* Even PR4 stated that what created some light for her was that she had to start *“to learn new skills and deliberately unlearn what was no longer adding value to her life*…*”* Thus, when a transcendent way of being is adopted, new meaning is facilitated when there is a shift from superficial reflection to a deeper reflexive form of thinking. The perceived distance from the self appears to open up new avenues of thinking and inquiry into the self and how it is being experienced in the moment.

In addition to creating perspective, ST appears to create a potential shift from being self-conscious to other-consciousness. One becomes aware that there is more out there. Middle managers unanimously reported that to succeed in the role as manager, they had to reach out to their staff to support them during the pandemic. In doing so, their employees would be able to attain their objectives and the business in turn would reap the benefits of their success. Respondent PR1 indicated that he had come to the realization that he had to *“*… *be of service to family, friends and employees*…*”* Another one (PR2) reported: *“*… *having a generous spirit is what sustained me during these difficult times*…*”* Similarly, PR5 drew from his Afrocentric worldview when he explained: *“*… *in African society, religion, morality and a sense of community play a significant role in the determination of one’s wellbeing. Thus, in the context of the pandemic, an individual’s loss is not theirs alone but that of the entire community. This affiliation is demonstrated through the notion of “kuchema pamwe,” which means, “mourning together.”* This deep sense of sharing in the loss and pain of others, is an appropriate example of what it means to be less egocentric and more other-conscious. This sense of community was also alluded to by another participant (PR7) as she recalled: *“*… *As a manager, I was forced to reach out to my staff. I had to put protocols in place to protect our employees, our clients and myself. I had to offer solutions to very novel challenges. My leadership authority compelled me to act and to act decisively in the interest of others*… *In hindsight, and in a sense this orientation provided me with a purpose*…*”*

The narratives of the middle managers dominated the theme of being other-conscious during the pandemic. Equally, PR4 narrated the following lived experience: *“I have implemented additional measures to ensure our employees can still access the counselors of our Wellness Programme. We have made additional methods of counseling available that will accommodate employees and their families who are not allowed to leave the house. These arrangements will ensure emotional support during a period in which employees may feel uncertain about their future at the company and perhaps even lonely*…*”*

In another narrative, PR3 responded that: *“I must do what I can to be of help to others.”* In its other-orientation, ST also seems to create social wellbeing. In a narrative by PR5 he focuses on the advantage of other-consciousness on his immediate family. With indirect relevance to the focus of this study, he details how the pandemic has influenced his family dynamics by sharing the following: *“My social cluster includes my wife and kids. This part of wellbeing has been strengthened as we get to spend more time together, play together and watching TV together. We’ve converted one of the TV rooms to what we call a camp site. This allows us to sit together until very late and sleep in one place.”*

This theme reflects how ST enables the facilitation of meaning in the moment, through the role of middle managers and by drawing on the support of a higher deity. In addition to the negotiation and re-negotiation of meaning, a number of potential shifts seem to emerge as they take independent deliberate action. Self-transcendence thus appears to be a resource for participants in terms of positive adaptation, by becoming work-oriented, reflexive and other-centered. This orientation provides them with the agency to be industrious, resilient and purpose-driven.

### Self-Transcendence Enables the Exploration of the Adaptive Benefits of Anxiety

Middle managers unanimously report that the pandemic was an anxiety-provoking experience and that this energy had driven them to adopt an other-directed orientation. For example, PR1 reports how despite being anxious, reaching out to others has paradoxically restored his sense of control in life by working in a more supportive and appreciative way with his colleagues: *“I could see the appreciation with which they responded to a simple phone call*… *I did not always have an answer, but I was there (trembling) to listen to the challenge*…*”* Other examples of working through the anxiety were mentioned: (PR9) *“*… *identifying opportunities to relearn, unlearn and learn new skills*…*”* PR5 said *“*… *I had to learn about online/virtual meetings which presented its own set of new challenges*…*”* Similarly, PR8 explained: *“*… *My anxiety drove me to spend time doing extra preparation to be fully prepared*…*”* PR7 also said: *“*… *I had to work proactively to manage the work and home environment to ensure that there will be no embarrassing disturbances from hubby and the kids*…*”*

Sometimes, something as engrained as a management style also had to change, despite the anxiety associated with this change, as narrated by PR3: *“*… *now I had to learn to manage staff through technology*…*”* Thus, the pandemic became an anxiety-machine. However, these middle managers were able to harness the adaptive benefits of anxiety. Some struggled to make sense of the perceived paradoxes; for example, *“how can I isolate, but we must fight the pandemic together?”* And again *“I care about you, but I cannot trust that I can come close to you*…*”* Despite being *“anxious, stressed and afraid, yes very afraid*…*”* as described by PR2, she further explained that life had to carry on, because *“employees needed answers, kids needed help with their homework, hubby needed food for his stomach, and I had to deal with all of this regardless whether I was anxious or my explanation that this is also my first COVID-19 pandemic!!!”*

This theme describes how the other-directed orientation as exhibited through ST has the capacity to enable participants to explore the adaptive benefits of anxiety as experienced by them during unique personal circumstances. When one experiences anxiety from a self-directed orientation, it seems to have a paralyzing effect. The focus is on self-preservation. However, when it is experienced from an other-directed orientation, it could have a liberating effect. The focus is then on being of service to others.

## Discussion

This qualitative hermeneutic phenomenological study set out to explore the impact of the COVID-19 pandemic on the meaning middle managers attach to their work by investigating the role of ST in cultivating meaning and wellbeing in a cohort of South African middle managers.

The findings highlight how ST could serve as coping mechanism during adversity. It also describes how ST facilitates the re-negotiation of meaning, resulting in potential shifts in disposition from a blame to a work orientation, reflection to reflexivity and self-consciousness to other-consciousness. The study also stresses how ST enables the exploration of the adaptive benefits of anxiety. Self-transcendence refers both to the movement beyond one’s immediate self-boundaries, and to a quality that emerges because of this movement, culminating in an expanded worldview (Garcia-Romeu, 2010, p. 26). Cloninger et al. (1993), conceptualize ST as “the extent to which a person identifies the self as … an integral part of the universe as a whole” (p. 975). The role of ST through its other-centeredness was evident in how participants were able to derive a sense of purpose from the way in which they focused on the other. Through their purpose-driven activities, participants were then able to cope with their personal challenging circumstances and became filled with a sense of meaningfulness. When we are engaged in meaningful work, it seems to have a positive impact on our wellbeing (Bakar et al., 2018; Kun and Gadanecz, 2019). Being stretched in adversity seems to create new meaning; for example, suffering contains an adaptive benefit, because one’s personal discomfort creates a positive difference in somebody else’s life. Furthermore, ST appears to have a spiritual dimension (Wong, 2016) because one tends to see beyond the obvious and material to experience the world as complex and dynamic when utilizing this spiritual vision. In doing this, hope, optimism and resilience can be created, because balance is restored in this dialectic experience (Koenig, 2020). Self-transcendence, therefore, appears to be a coping resource in terms of positive adaptation. In becoming work-oriented, reflexive and other-centered, this other-directedness creates the necessary agency for individuals to be more diligent, resilient and purpose-driven.

From the experience of participants, it is evident that work is both a subjective and objective experience (Bartels et al., 2019) and it is through work that we search for being, for meaning and a sense of belonging (Deci and Ryan, 2008). Work, therefore, is critical to our personal and social integration (Ransome, 1996) and connects us to others. Participants reported that identity and a coherent sense of self are developed through work located in each context (Person et al., 2016). What is relevant to this study is that through an other-directed orientation to work and life, purpose and identity are created because through subjective appraisals, meaning and significance are attached to specific work experiences (Rothmann and Hamukang’andu, 2013). Furthermore, meaningfulness and wellbeing are created in the dynamic interface between the self and the other, or other systems. Thus, ST (reaching out to others) seems to play a critical role in creating potential space for the creation of meaning and wellbeing (Wrzesniewski et al., 2013). By reaching out to others, we shape and influence the world through the realization and exercise of our personal agency and authority (Bartels et al., 2019). If work creates a sense of social connectedness (Ciulla, 2000; Beukes and Botha, 2013), ST seems to do even more to enhance our social connectedness, relationships and social wellbeing.

This study confirms that ST could also be conceptualized as the expression of and the creation of a sense of relatedness to others. Research reports that a perceived sense of belonging has a positive impact on experienced meaning in life (Lambert et al., 2013). The opposite is also true. A perceived sense of loneliness and isolation, as often experienced during the COVID-19 pandemic, has a negative impact on meaning in life (Hicks and King, 2009; Stillman et al., 2009). The study also asserts that ST could be associated with beneficence, which has been identified as an important predictor of wellbeing (Aknin et al., 2013). If various forms of beneficence are connected to a greater sense of meaning (Van Tongeren et al., 2015), then it can be concluded that ST insofar as it reflects a reaching out to others and contributes to something larger than oneself, could result in a meaningful life. Furthermore, acts of caring toward others and transcendence of one’s personal circumstances have been reported as a major source of meaning and wellbeing (Martela et al., 2018).

The outbreak of the coronavirus disease (COVID-19) has been experienced as a catastrophic calamity with a direct impact on people’s socio-psychological wellbeing and anxiety levels (Peteet, 2020). Many people throughout the globe are experiencing anxiety, including the aged, those with underlying health conditions, medical staff and even young people, given the nature of the second wave of the pandemic. In a recent study by Sundarasen et al. (2020) on the psychological impact of COVID-19 and lockdown among university students in Malaysia, it was found that stressors include financial pressure, the challenges of remote online teaching and uncertainty about the future regarding their academic performance and career prospects. Anxiety is therefore real and is conceptualized as an emotional state and/or the emotional and psychological reaction of the unconscious to threats in the external or internal world (Vansina and Vansina-Cobbaert, 2008). Anxiety can be characterized as paranoid (when one feels threatened) and persecutory (when one feels victimized; Sievers, 2009). Individuals feel that they must defend themselves against these anxieties as a form of containment and to retain control (Sievers, 2009). It has been reported that the current pandemic can trigger deeply rooted existential anxieties (Peteet, 2020). These anxieties could threaten our sense of belonging and sense of place in the world and trigger our need for self-preservation. However, this study has shown that ST, which is contrary to the act of self-preservation in adversity, has the potential to shift the individual beyond her/his immediate self-boundaries. This way of being results in a broadened revolutionary worldview and an identity grounded in the belief that we are all simultaneously intimately part of and integrally connected to our external world. Therefore, in the struggle of life, we are called to respond to the suffering of others. This is essentially a call to other-centeredness, which reverberates through the human quality of ST guided by a meaningful and mindful mindset (Siegel, 2010).

What should be pointed out at this stage of the discussion is that the tenure of the managers in their respective positions could have influenced the way in which they had experienced the pandemic and how they were able to respond to this form of adversity. The gender of the participants could also have had an influence on the nature of the narratives which were shared with the researcher. These are important considerations when exploring the phenomenological experiences of the participants.

## Conclusion

The COVID-19 pandemic has severely restricted employees in terms of the nature of their work and their need to interact with their colleagues. However, the pandemic also presented new opportunities in the form of being other-centered by reaching out to others. Crises often reveal and confirm identity. This is also applicable to the COVID-19 pandemic. This human and economic tragedy generally revealed the stewardship dimension of the pandemic. However, as the dialectical and dynamic nature of the pandemic evolved, it quickly exposed the dark underbelly of humanity. In the face of suffering and adversity, one should always be moved. Thus, suffering should generate an external response. The human response to suffering should be a shift from self-centeredness to other-centeredness. Self-transcendence, or other-centeredness is an affirmation that we cannot be immune to pain and indifferent to suffering. One of the contributions of this study lies in how the lived experiences of middle managers as expressed through their narratives reveal how being self-transcendent could facilitate meaning-making and serve as a coping mechanism during times of adversity, as characterized by the COVID-19 pandemic.

One of the practical implications of the findings is that reflexivity seems to be key in the context of creating a meaningful mindset and to enhance one’s sense of purpose and wellbeing. It is suggested that organizations invest in reflexive practices as a tool to promote deep learning and connectivity by exploring dialectical processes through reflexive work. Another practical implication is that organizations should also pay more deliberate attention to the nurturing of ST by striving to be purposeful in its activities, because this study affirms that ST plays a significant role in the building of psycho-social resilience, relatedness and character. Thirdly, ST appears to drive individual wellbeing and to restore our sense of control in life. There appears to be an association between ST and wellbeing. Thus, a sense of purpose restores some form of control in one’s life. Finally, ST seems to create potential space (between the self and other) to explore the adaptive benefits of negative emotions and experiences. Renewed possibilities and opportunities for engagement are created when focus on the self is reduced and focus on the other is enhanced.

The relevance of the identified sample, its hermeneutic design and the qualitative nature of the study enabled a rich, in-depth and contextual understanding of the research phenomenon. However, the study is limited in terms of generalization owing to the use of unstructured written narratives, which could have resulted in the unintended omission of salient data and the nature and constitution of the sample (gender bias), which also excluded the experiences of other socio-demographic groupings. Another limitation could have been the potential impact of the tenure of the managers in their respective managerial positions. Tenure could have had a significant impact on the phenomenological experiences of the participants. Other forms of data collection could have allowed for the exploration of specific aspects related to the study. Future research should explore each theme in more depth, as well as the resources required to adopt a unique other-directed orientation.

## Data Availability Statement

The raw data supporting the conclusions of this article will be made available by the authors, without undue reservation.

## Ethics Statement

The studies involving human participants were reviewed and approved by University of South Africa IOP Ethics Review Committee. The patients/participants provided their written informed consent to participate in this study.

## Author Contributions

The author confirms being the sole contributor of this work and has approved it for publication.

## Conflict of Interest

The author declares that the research was conducted in the absence of any commercial or financial relationships that could be construed as a potential conflict of interest.
